# Enhancing Tumor Detection in IR-UWB Breast Cancer System

**DOI:** 10.1155/2017/4606580

**Published:** 2017-03-19

**Authors:** Sara Fouad, Reda Ghoname, Abd Elmonem Elmahdy, Abd Elhalim Zekry

**Affiliations:** ^1^The Higher Institute of Engineering, Modern Academy, Cairo, Egypt; ^2^The Electrical and Computer Department, University of King Abd El Aziz, Jeddah, Saudi Arabia; ^3^The Electronics and Communication Department, Faculty of Engineering, Ain shams University, Cairo, Egypt

## Abstract

An ultra-wideband (UWB) microwave system for breast cancer detection is presented. The proposed system includes monocycle pulse generator, antipodal Vivaldi antenna, breast model, and calibration algorithm for tumor detection. Firstly, our pulse generator employs transmission gate in glitch generator to achieve several advantages such as low power consumption and low ringing level. Secondly, the antipodal Vivaldi antenna is designed assuming FR4 dielectric substrate material, and developed antenna element (80 × 80 mm^2^) features a −10 dB return loss and bandwidth ranges from 2.3 GHz to more than 11 GHz. Thirdly, the phantom breast can be modeled as a layer of skin, fat, and then tumor is inserted in this layer. Finally, subtract and add algorithm (SAD) is used as a calibration algorithm in tumor detection system. The proposed system suggested that horizontal antenna position with 90° between transmitting and receiving antennas is localized as a suitable antenna position with different rotating location and a 0.5 cm near to phantom. The mean advantages of this localization and tracking position around breast is a high received power signal approximately around mv as a higher recognized signal in tumor detection. Using our proposed system we can detect tumor in 5 mm diameter.

## 1. Introduction

Recently, microwave imaging in task of detection and location of malignant tissue in the woman breast has received a considerable amount of interest [1–6]. A microwave imaging system is considered as a viable alternative to X-ray mammography due to its several advantages such as cost and insignificant side effects. Microwave imaging includes the propagation of very low levels of microwave energy through the breast tissue. Normal and malignant breast tissue have a difference in the electrical properties as the basis for tumor detection and location.

Normal breast tissue is relatively translucent to microwave radiation, while the malignant lump contains more water and blood, leading to microwave signal backscattering. This scattered signal can be picked up by a microwave antenna and can be analyzed using an image processing to handle on computer [5]. The basic idea of breast cancer detection is to send ultranarrow low power pulses to the patient's body and make a decision depending on the received signal by comparing it with an average of backscattered signals, or a signal backscattered from normal tissues.

The pulse generator is a main block in an imaging system. It has been designed using ultra-wideband impulse radio (IR-UWB) technology [7]. IR-UWB is a very short energy pulse, typically on the order of a hundred picoseconds transmitted with the use of an antipodal Vivaldi designed wideband antenna.

Digital IR-UWB pulse generation [8, 9] has been widely studied as a new approach to achieve low power characteristic. IR-UWB deployment in the medical applications is highly desirable, as there are different advantages, such as higher data rates, lower power dissipation, and enhanced medical security to the patient.

The second element on radar techniques is based on the use of ultra-wideband (UWB) antennas, in which a short pulse is transmitted into the body; then the reflected signals are detected by one or more antennas at a receiver placed in different locations [10]. The antipodal Vivaldi antennas (APVA) presented in the literatures [10, 11] satisfies the requirements for imaging systems such as bandwidth, gain, and impulse response albeit at the expense of the size of a large volumetric. Designing UWB transmitter including pulse generator and antenna is proposed in this paper and then used in tumor detection. First the pulse generator (PG), with the stringent constraints of small area, low ringing, and low power consumption, is designed and implemented. Second the APVA which meets the above-mentioned requirement is developed. Finally, transmitting pulses generated by a proposed pulse generator are made to illuminate a cancerous breast model, with backscatter signals that is received by the receiver antenna placed around the cancerous breast. The cancerous tissue significantly affects the received signal due to a high dielectric difference between malignant and healthy tissue.

The paper is organized as follows; the new design of IR-UWB pulse generator is presented with an output monocycle pulse at 4.2 GHz center frequency compatible with breast cancer imaging system. The design, implementation, and results, using cadence tool, are also presented in Section 2. In Section 3, an Antipodal Vivaldi antenna (APVA) is designed and fabricated on a FR4 with *ε*_*r*_ = 4.5, and *h* = 1.5 mm. It covers the required UWB band from 2.3 to more than 11 GHz and features directive properties. The system is completed with spherical breast phantom and a receiving APVA. The impact of tumor presence on the received signal and operational conditions are optimized in Section 4. In Section 5, an artifact removal algorithm is described to eliminate the large reflections from the skin and the incident signal. Finally Section 6 concludes the paper.

## 2. Monocycle Pulse Generator

Monocycle pulses [12] are preferred to simple pulses because they have no dc components, which could represent a limit for the spectral mask compliance and radiation antenna efficiency.

Analog filtering is the simplest one of monocycle UWB pulse generator methods. A common way of directly generating an UWB pulse without using a carrier is to first form a baseband impulse with very short time duration glitches and a high frequency bandwidth and then filter the glitches using a band-pass or a pulse shaping filter. The conventional glitch generator is based on pulse delay element such as inverters to delay pulses by specific delay value and then combining the delayed and the original pulses by an AND gate. In our design the transmission gate is used instead of NAND gate to produce a monocycle pulse of UWB with comparable low power, good balance between the positive and negative parts, and relatively small ringing level. This pulse generator is named transmission gate pulse generator (TGPG).

### 2.1. Pulse Generator Design

**Figure 1:**
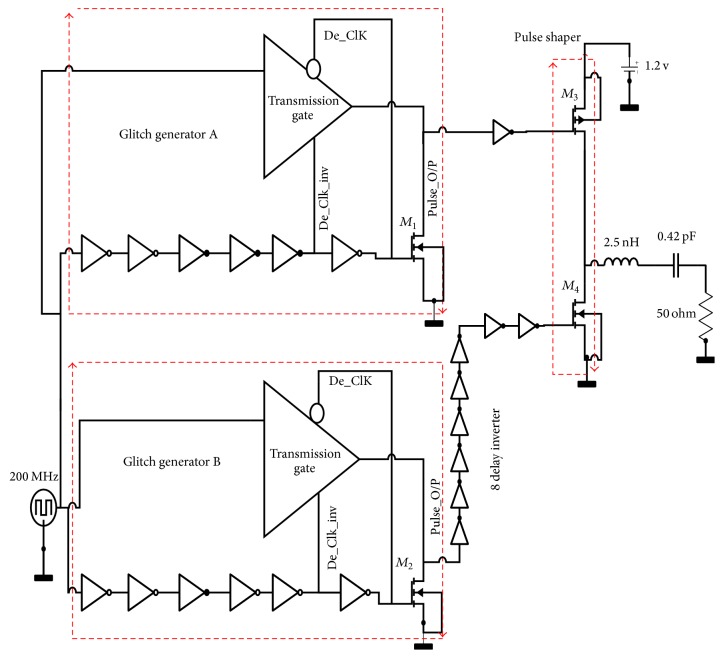
The new design of IR-UWB pulse generator.

A transmission gate pulse generator (TGPG) based on glitch generation [13] is proposed in this work to produce a monocycle pulse. Two identical glitch generators named GGA and GGB are the back bone of pulse generator circuit as shown in Figure 1. The proposed TGPG consists of transmission gate controlled by two delayed clock pulses De_clk, De_clk_inv, and its positive glitch output connected to drain of *M*_1_ and *M*_2_.

There are two cases observed in our design; the first case is a rising clock edge in which De_clk equal “0” and De_clk_inv equal “1.” The corresponding data is transferred to drain of *M*_1_ and *M*_2_ (node pulse_o/p) as the transmission gate is ON. After 6 *τ*_inv_ delay, delay between main clock and (De_Clk), the NMOS *M*_1_ and *M*_2_ turn on and discharge the node Pulse_O/P to ground so the Gaussian pulse is designed. The second case is falling clock edge (De_clk equal “1” and De_clk_inv equal “0”) in which node Pulse_O/P is connected to ground via NMOS *M*_1_ and *M*_2_ at the primary time 6 *τ*_inv_. Afterwards, the transmission gate turns on and keeps the node Pulse_O/P connected to “0.”

Then the output of the glitch generator is used to derive a pulse shaper consisting of two CMOS transistors and a shaping filter in form of a resonance circuit. It is an off chip filter where the inductor is a bond wire while the capacitor is added to complete the resonance circuit and the resistor is that of the load. As clear from the schematic in Figure 1, the GGA output is inverted and applied to the pull up PMOSFET *M*_3_ of the output pulse shaper; as a result, the pulse generator output at the drain of the output transistors becomes *V*_DD_. The output glitches of GGB are delayed using eight-stage inverter chain and applied to the pull down transistor NMOSFET *M*_4_ that drives the output voltage to 0 V and generates the negative pulse.

The sizes of output pulse shaper stage transistor *M*_3_ and *M*_4_ have to be selected carefully for correct pulse shape. During the design procedure, the supply voltage is kept constant at its nominal value of 1.2 V. The gate sizes for the building blocks are determined to have a UWB pulse 235 ps. The transmitter output after pulse shaper transistors is applied to the package and off chip printed circuit board (PCB) through a wire bond that can be considered as an equivalent to inductance *L*_wb_ 2.5-nH as shown in Figure 1 [12]. A 4.2 GHz-band BPF is realized using a 0.42 pF series capacitance *C*_ps_ with *L*_wb_.

**Figure 2:**
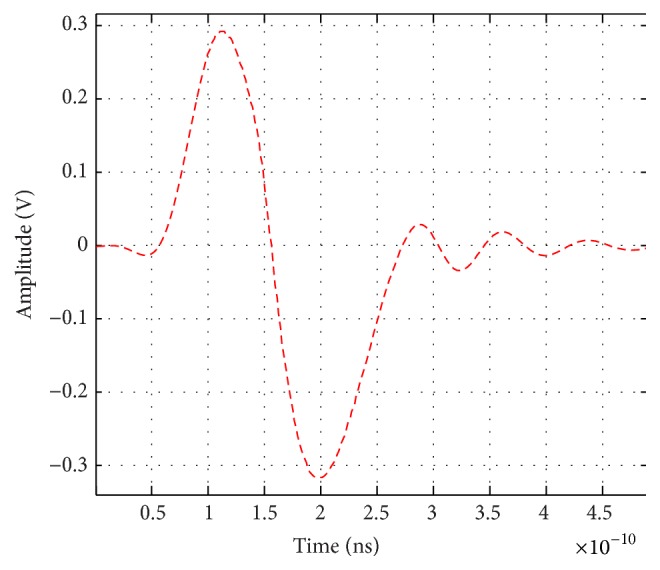
Proposed monocycle pulse with 235 ps pulse duration operating at ≈4.2 GHz as center frequency.

The proposed monocycle pulses with 235 ps pulse duration and approximately operating at 4.2 GHz as center frequency are shown in Figure 2. A Gaussian monocycle of 235-ps width is obtained after PEX practices extraction test. The peak-to-peak voltage is 600 mV, and it has good balance between the positive and negative parts, and it has relatively small ringing level.

### 2.2. Implementation Results

**Figure 3:**
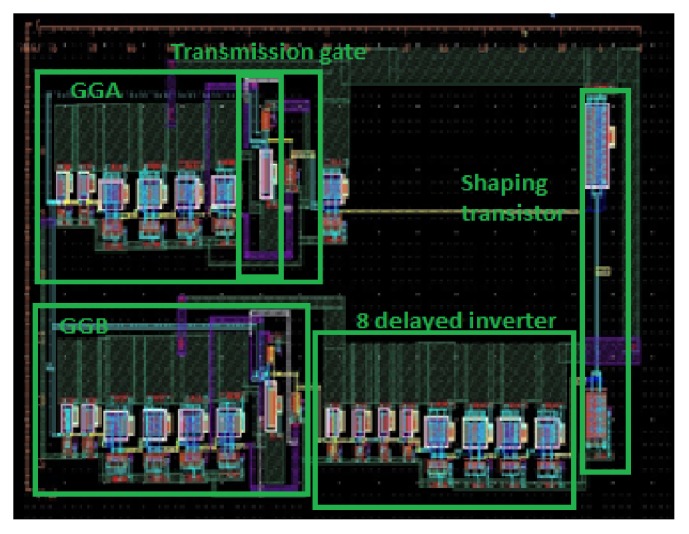
Layout of monocycle pulse generator.

The proposed transmitter is designed and simulated using cadence virtuoso and UMC MMRF 130 nm CMOS process. The layout is shown in Figure 3 after DRC (Design Rule Check) and LVS (Layout Versus schematics). The whole transmitter including the glitch generator and buffers occupies only a rectangular area of 76.69 *μ*m × 37.15 *μ*m.

Slow-Slow (S-S), Fast-Fast (F-F), and Typical-Typical (T-T) of NMOS and PMOS performance tests at different temperatures *T* = 27°, −20°, and 70° are performed for the pulse generator to see if the generated pulses are still usable for breast cancer imaging or not.

**Figure 4:**
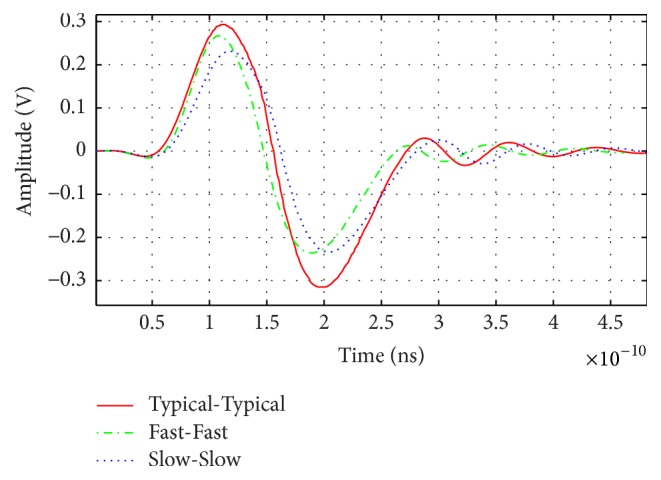
Typical-Typical, Fast-Fast, and Slow-Slow simulations.

The variation of the amplitude and the duration of generated pulses after Practices Extraction PEX test are shown in Figure 4 for the different operating temperatures, supply voltage, and different fabrication tolerances. The peak-to-peak voltage and pulse duration of each case are recorded at different temperatures in Table 1.

Under T-T test with *T* = 27°, and *V*_dc_ = 1.2 V, the duration and amplitude follow the characteristic of monocycle pulse. Slow-Slow test expanded pulse duration to 250 ps meaning a center frequency of 4 GHz at *T* = 70°, and *V*_dc_ = 1.1 V, but for Fast-Fast test the duration changed to 200 ps with a corresponding center frequency of 5 GHz at *T* = −20°, and *V*_dc_ = 1.3 V. These measured values are given in Table 1. From the previous results we conclude that this design has a low ringing level and compatible to breast cancer imaging system.

Comparison between the performance parameters of our PG and the previous UWB pulse generators [12, 14–16] presented in Table 2. Our static power consumption is calculated by cadence tools. It is seen that our UWB pulse generator has lower power consumption than all previous works. Note also that this design exhibits one of the highest pulse peak-to-peak amplitude on standard load impedance, low static power consumption, and low level in ringing effect.

## 3. Antipodal Vivaldi Antenna

Antipodal Vivaldi antenna is a type of tapered slot antenna which is an end fire antenna. The antenna consists of the feeding line and the transition and the radiating structures. In general, the radiated structure is exponentially or elliptically tapered, which means that they are composed of two layers structure [10, 17].

Antipodal Vivaldi antennas presented in the literature [18, 19] satisfies the requirements for breast cancer imaging systems in terms of bandwidth, gain, and impulse response albeit at the expense of significant volumetric size.

**Figure 5:**
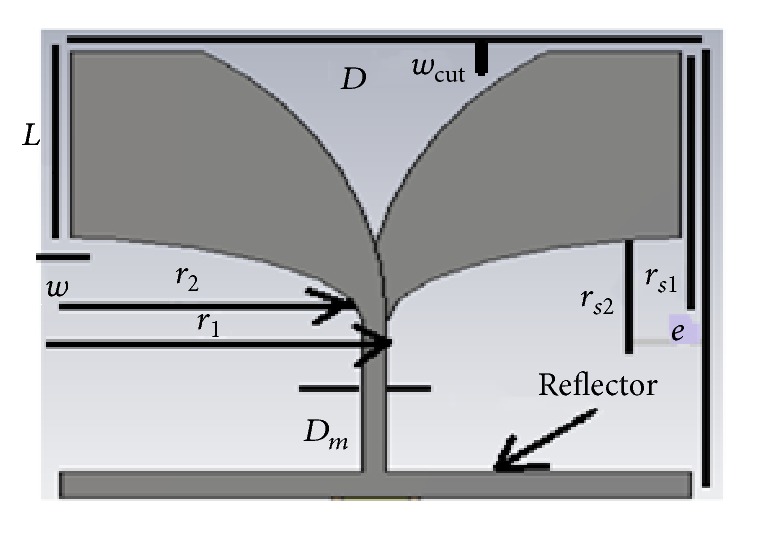
Configuration of the proposed antipodal Vivaldi antenna.

The proposed antipodal Vivaldi antenna for inclusions in an UWB microwave imaging system [10] is shown in Figure 5. The design objective is to obtain its bandwidth requirement of 2.3 up to 11 GHz. The following design procedure is utilized in designing the proposed antipodal Vivaldi antenna:

The width *D* and length *e* of the antenna structure, excluding the feeder, could be calculated from the following equation with the lowest frequency of operation *f*_*l*_, thickness of the substrate *h*, and dielectric constant *ε*_*r*_ [10].

(1) $$\begin{array}{c} D=e=\frac{C}{f_{l}}\sqrt{\frac{2}{\varepsilon_{r}+1}}, \end{array}$$

where *C* is the speed of light in free space.

Then, the radiating structure of the antenna is designed from the intersection of quarters of two ellipses as depicted in Figure 5. The major radii *r*_1_ and *r*_2_ and the secondary radii *r*_*s*1_ and *r*_*s*2_ of the two ellipses are chosen according to the following equations:

(2) r1=D2+Dm2r2=D2−Dm2rs1=e−wcutrs2=0.48r2,

where *w*_cut_ is used to control the lowest frequency of operation.

The microstrip transmission line feeder has a width of *D*_*m*_ and dielectric thickness *h* to give the characteristic impedance, *Z*_0_ = 50 ohm that can be calculated using the equation

(3) $$\begin{array}{c} D_{m}=\frac{120\pi h}{\sqrt{\varepsilon_{r}}Z_{0}}. \end{array}$$

The proposed design is verified using the commercial software package, CST microwave studio, and experimental tests.

### 3.1. Measurement Results

**Figure 6:**
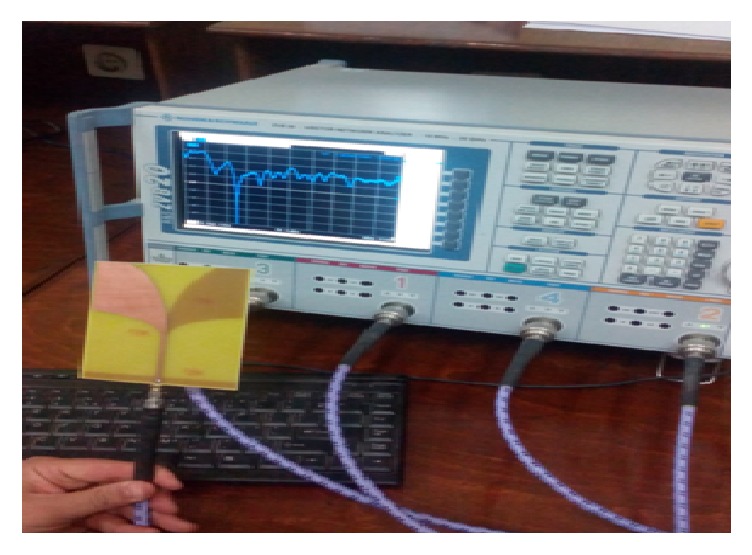
Measuring set up of the return Loss of fabricated antenna.

**Figure 7:**
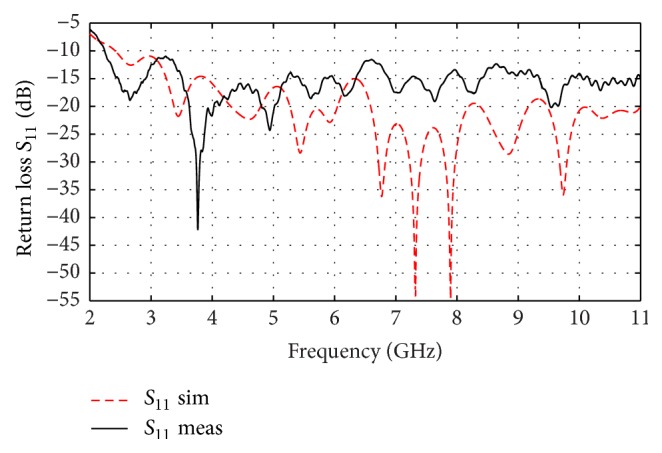
Simulated and measured return loss of proposed APVA (parameters *D* = *e* = 80 mm, *r*_1_ = 43.2 mm, *r*_2_ = 40 mm, *D*_*m*_ = 3.2 mm).

Figure 6 is a photograph showing the antenna and the test setup.  Figure 7 shows the simulated and measured return loss of the antipodal Vivaldi antenna developed in FR4 (*ε*_*r*_ = 4.5, *h* = 1.5 mm) material.

**Figure 8:**
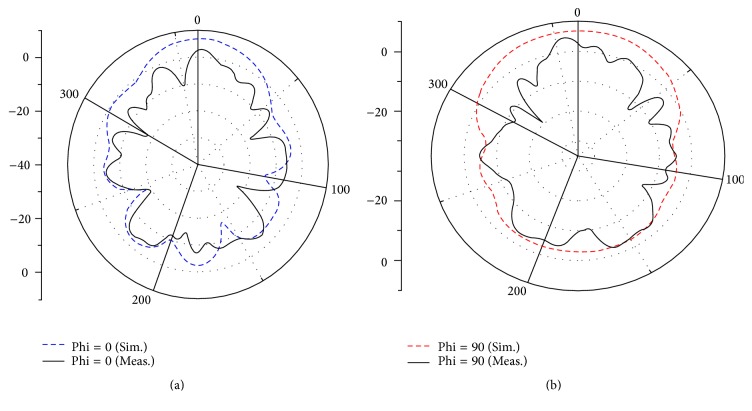
Normalized radiation pattern of the proposed antenna at 4.2 GHz. (a) E-plane and (b) H-plane.

One can see from Figure 7 that the −10 dB return loss bandwidth extends from 2.3 to more than 11 GHz covering the required UWB band of 3.1–10.6 GHz. The measured and simulated results are similar validating the antenna specifications. Measured and simulated far-field radiation patterns are shown in Figure 8 in E-plane and H-plane at 4.2 GHz. The measurement is made in an anechoic chamber. Acceptable agreement is found between measured and simulated radiation pattern.

## 4. UWB Microwave Imaging for Breast Cancer Detection

The breast is largely transparent to microwave radiation for imaging. Significant, electromagnetic property contrast may appear between healthy and tumors tissues [20].

Electrical properties of tissues at microwave frequencies have been extensively studied for dosimeter, therapy, and diagnostic applications. The electrical or dielectric properties include relative permittivity (*ε*_*r*_) and conductivity (*σ*). Water is a key factor in determining tissue permittivity [21]. Low water content tissues, such as fat, have lower permittivity values than high water content tissues such as muscle and skin [21]. As described in electrical properties of tissues, less attenuation and reflection are expected from normal tissues than tumor tissues. Radar-based approaches to breast tumor detection indicate the location of strongly scattering objects, rather than creating maps of the distributions of electrical properties.

There are several different approaches to UWB radar imaging. These approaches can be divided into three categories: monostatic, bistatic, and multistatic. In the monostatic case, the transmitting antenna itself acquires the backscattered signal. Often the transmitting antenna is shifted across the breast to make a synthetic aperture. In the bistatic configuration, two antennas are used, a single transmitting antenna and a single receiving antenna. At last, in the multistatic approach, the tissue is illuminated by one transmitting antenna while the backscattered signals are recorded at several antennas placed at different positions around the breast [22].

### 4.1. Breast Model and Investigation of Antennas Angular Direction

**Figure 9:**
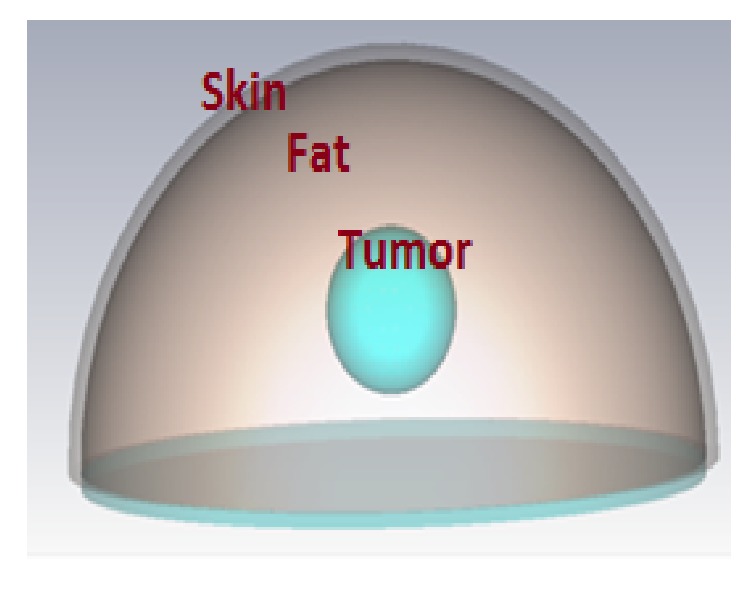
Breast model with tumor.

For the hemispherical system, the breast is modeled as a half space of sphere. The breast model is a 10 cm diameter sphere with height 6 cm surrounded by a 2 mm thick layer of skin (Figure 9). The materials in the simple breast models (indicated in Figure 9) are assigned appropriate electrical properties. The dielectric properties of normal breast tissue are assigned random variations of up to 10% around nominal values of *ε*_*r*_ = 9 and *σ* = 0.4 S/m, distributed over full sphere. The assumed contrast between malignant and normal breast tissue is approximately 5 : 1 in relative permittivity and 10 : 1 in conductivity [23]. The skin is assigned the following values: *ε*_*r*_ = 36 and *σ* = 4 S/m. The tumor is modeled as a sphere inside the fat with 2 cm diameter. The dielectric constant and conductivity for tumor tissue are assumed to be *ε*_*r*_ = 50 and *σ* = 4 S/m, respectively, over the microwave frequency band.

In the proposed system the Gaussian monocycle pulse (GMP) is sent to transmitter antenna and then illuminates the breast phantom model, and the receiver antenna collects the backscattered waves from breast phantom model. In order to achieve a higher level amplitude of signal for enhancement the tumor signal, the end fire direction for antenna position is introduced.

**Figure 10:**
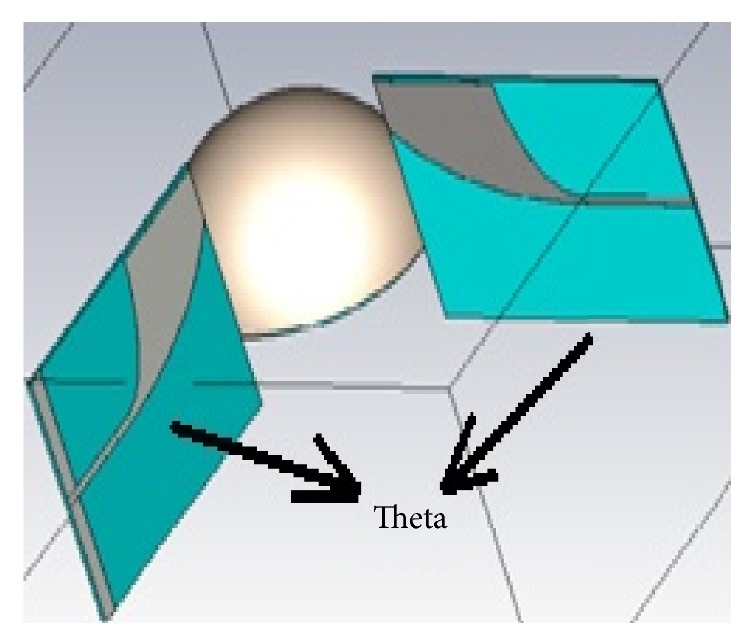
Vertical end fire antenna position.

Antipodal Vivaldi antenna is located at different end fire position (vertical and horizontal end fire) as shown in Figures 10 and 12 to get the appropriate position. To decide what is the position that leads to collection of a high power signal at received antenna *S*_21_ parameters of both vertical and horizontal end fire antennas are compared. *S*_21_ Parameter becomes an acceptable value in tumor detection if it rounded off −30 dB.

**Figure 11:**
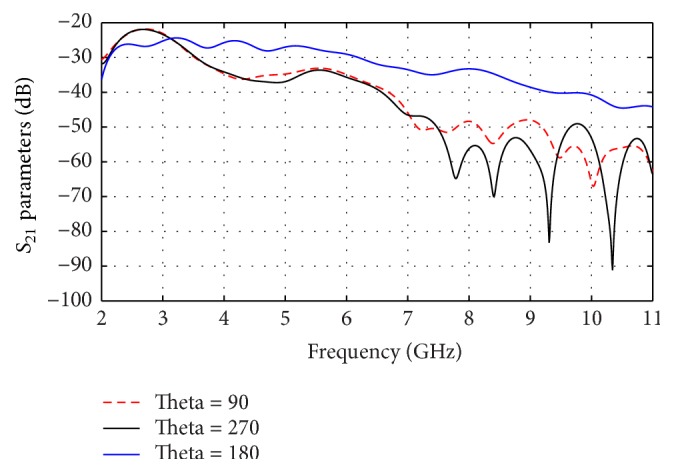
Effect of angles between antennas for vertical end fire on *S*_21_.

**Figure 12:**
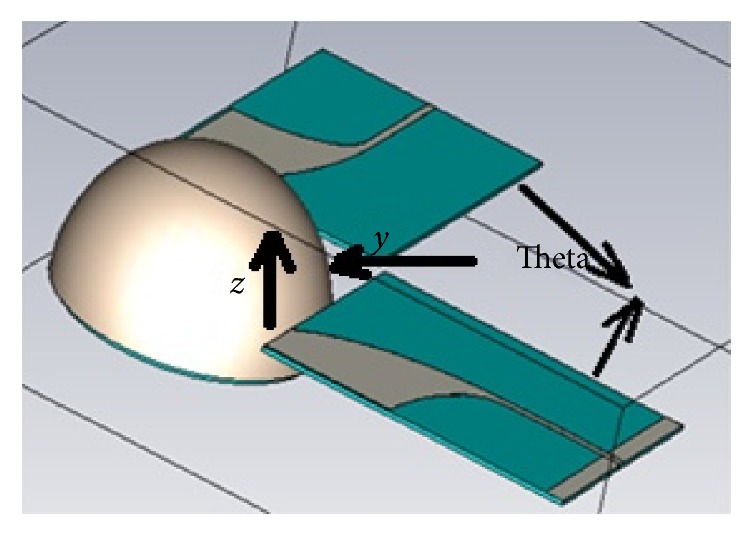
Horizontal end fire antenna position.

The calculated *S*_21_ curves of vertical end fire antenna are shown in Figure 11 for separation angles of 90°, 180°, and 270° between transmitter and receiver antennas, respectively. As observed that the higher power (e.g., −25 dB) at 180° is achieved from 2.3 to 6 GHz but out of this range of frequency the power is decreased to, for example, −45 dB.

**Figure 13:**
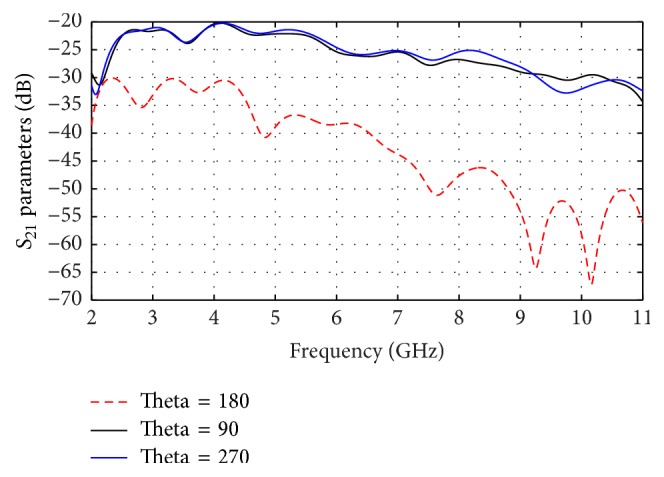
*S*_21_ for horizontal end fire at different angles between the two antennas.

The simulated *S*_21_ curves of horizontal end fire antenna are shown in Figure 13 at the angles 90°, 180°, and 270° between transmitter and receiver antennas. As observed that the higher power (e.g., −20 dB) at 90° and 270° is achieved from 2.3 to 6 GHz but out of this range of frequency the power is decreased to, for example, −30 dB, which still acceptable.

From previous result we conclude that horizontal end fire antenna position with 90° and 270° between transmitting and receiving antennas is an optimum configuration for enhancing a process of tumor detection. In this work we chose horizontal end fire antenna position with 90°.

### 4.2. Spacing between Antennas and Breast Surface

Now the effect of horizontal antenna separation away from the breast in *y* direction is studied.

**Figure 14:**
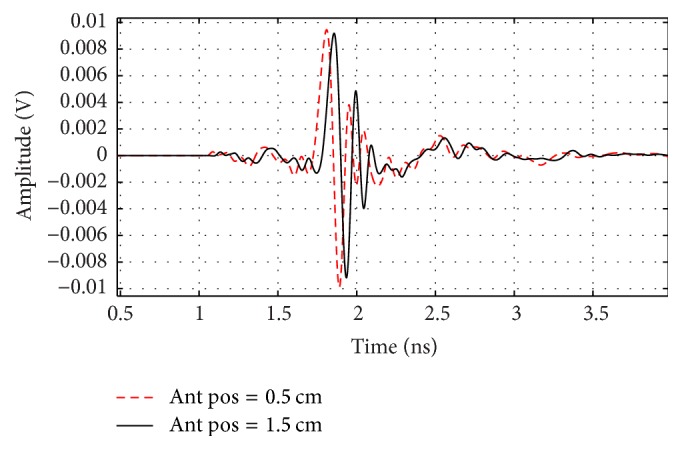
Output signals for *y* = 5 and 15 mm antenna positions.

**Figure 15:**
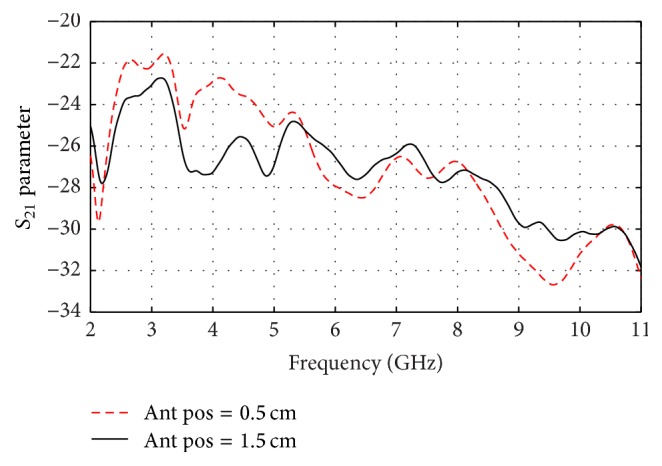
*S*_21_ Parameter for 5 and 15 mm antenna location in *y* direction.

The received signals and *S*_21_ parameters are shown for two separation processes of 5 and 15 mm in Figures 14 and 15, respectively. It is clear that the received signal at 15 mm is weaker and delayed with respect to the received signal at 5 mm which is an expected result. Also *S*_21_ is greater at the lower frequencies at the 5 mm distance than that at the 15 mm.

### 4.3. Detection of Tumor at Breast Model and at Different Radii

**Figure 16:**
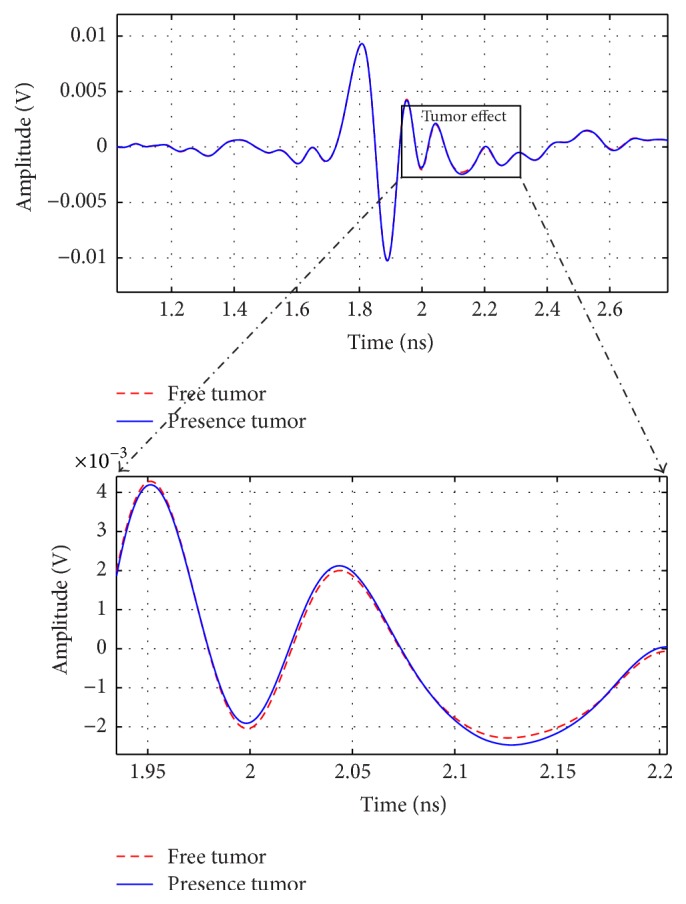
Received signals due to reflection from tumor free breast and with tumor breast.

A breast phantom model is located between transmitter and receiver antennas and illuminated with the proposed monocycle pulse. Output signal has high amplitude for the suitable horizontal end fire antenna with 90° between the two antennas as shown in Figure 16. So, detection of the tumor can be recognized (e.g., from 1.9 to 2.6 ns) at the received signal with signals in the level of mv which is three decades higher than that observed in [24].

**Figure 17:**
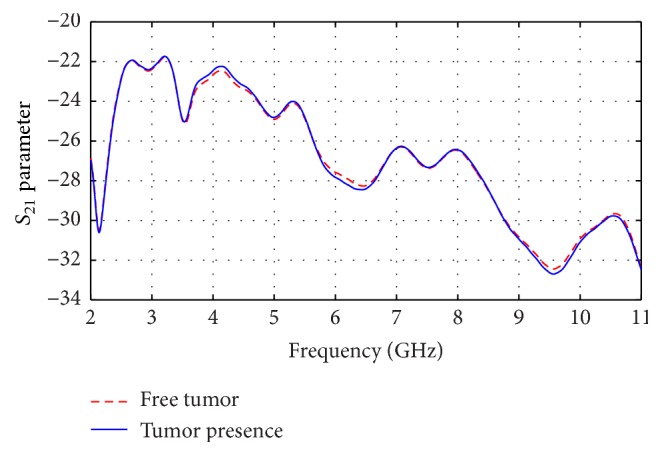
*S*_21_ at both cases: free of tumor and presence of tumor breasts.

*S*_21_ curves of both cases: presence of tumor and free of tumor breast are shown in Figure 17. We observed that tumor effect could be recognized.

**Figure 18:**
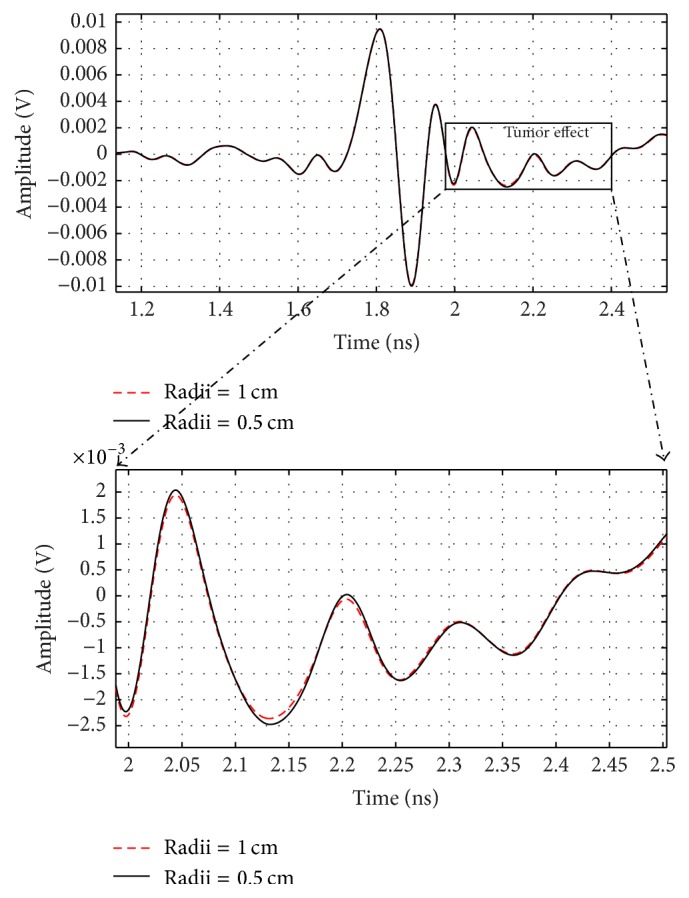
Output signals for 5 and 10 mm tumor radius.

When changing tumor radius to be 5 and 10 mm in the breast model, the received signal changes as shown in Figure 18.

## 5. Tumor Detection and Calibration Approach

It is seen from the previous tumor simulation experiments that the received signal with tumor is basically different from the signal without tumor. However the difference is not appreciable such that one cannot easily judge for the presence of tumor. Therefore a special scan scenario and signal processing are needed to clearly identify the tumor.

### 5.1. Preprocessing for Artifact Removal

It is found that the recorded backscattered signals consist of two parts: the early-stage and the late-stage. The majority of early-stage parts consist of incident signals and strong reflections from skin fat interface. Meanwhile the late-stage parts include tumor response, fatty tissue response, and the multireflections between these tissues. The skin, fatty, and tumor responses indicate that the signals directly reflected from these tissues. For identification, only tumor response is needed, and all other signals are viewed as superposition early-stage artifact and the late-stage clutter [25]. These artifacts can be several orders of magnitude greater than the desired tumor response; thus they must be removed before applying any image reconstruction algorithm.

**Figure 19:**
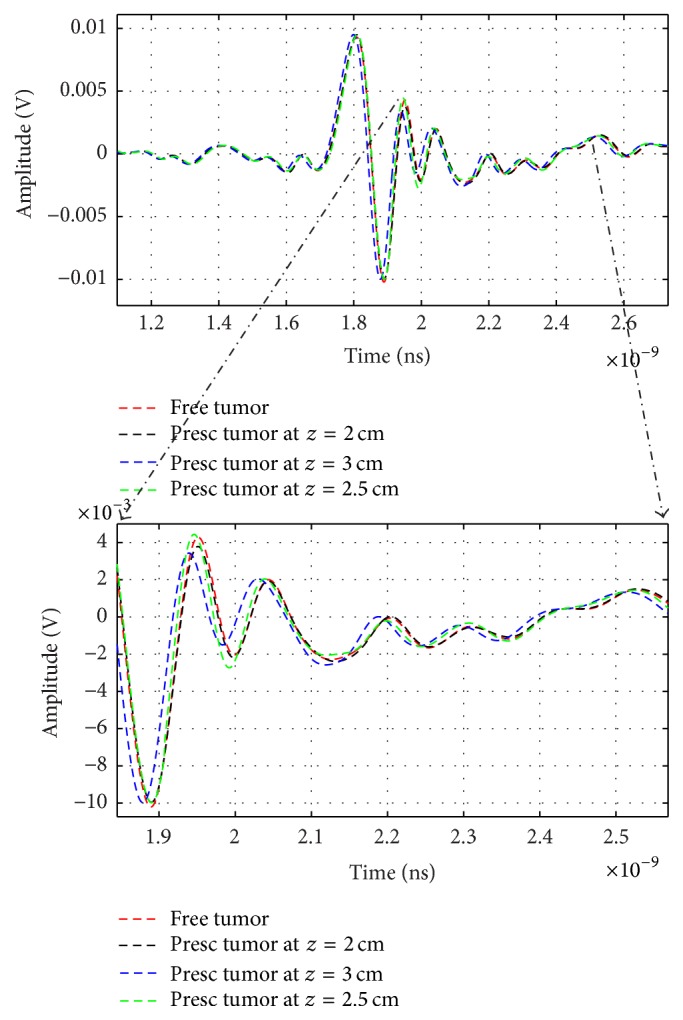
Output signals for *z* = 2, 2.5, and 3 cm antenna positions.

In our breast cancer imaging system horizontal end fire antennas are placed with 90° between transmitted and received antenna at 5 mm apart from skin fat interface. These antennas are moved together for *z* = 20, 25, and 30 mm.  Figure 19 illustrates the shape of received signals at different antenna position. Calibration steps are depending on the assumption that the signals recorded at different antenna locations have similar incident pulse and skin backscatter content [25]. The tumor signal is different in amplitude and time as a result of multipath propogation and different tumor antenna distance. This variation is observed (e.g., from 1.8 to 2.6 ns) as the tumor response is observed.

### 5.2. Analysis of Artifact Removal and Tumor Detection

In order to remove the artifacts and extract the tumor signal one used a calibration algorithm called subtract and add, SAD algorithm. In the first step of the algorithm one subtracts the signals between two adjacent locations and second step is summed subtract signal from adjacent location at the same time instant.

Assuming that the received signal from the breast at position *z* is *S*_*i*_(*t*) and at the position *z* + Δ*z* is *S*_*i*+1_(*t*), then the tumor signal *S*_*T*_*i*__(*t*) can be expressed by difference [26].

(4) $$\begin{array}{c} S_{T_{i}}\left(\;t\;\right)=S_{i+1}\left(\;t\;\right)-S_{i}\left(\;t\;\right). \end{array}$$

By repeating the measurements at *n* × *z*-locations and building the difference between two neighboring locations, one can fortify the tumor identification signal by summing all the difference. Then the final tumor signal *S*_tumor_ can be expressed by summing all differences *S*_*T*_*i*__ from *i* = 1 to *n*.

(5) $$\begin{array}{c} S_{tumor}\left(\;t\;\right)=\overset{n}{\underset{i=1}{\sum }}S_{T_{i}}\left(\;t\;\right). \end{array}$$

**Figure 20:**
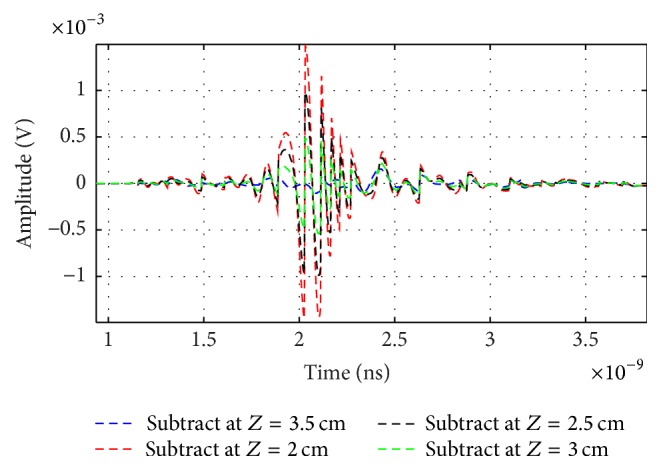
Output from subtract steps at different antenna locations.

Figure 20 shows the tumor signals *S*_*T*_*i*__(*t*) at different locations *z* from 2 cm to 3.5 cm in steps of 0.5 cm. It is clear that the different subtracted signals are appreciable and in the order of mV.

**Figure 21:**
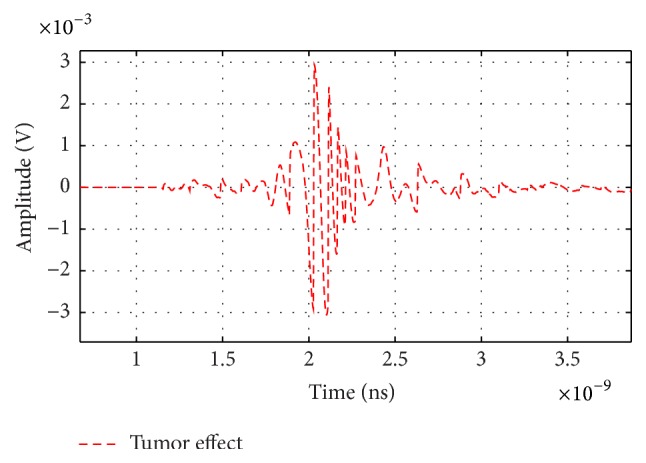
Tumor signal summing from different antenna locations.

Figure 21 depicts the overall accumulated tumor signal resulting from the all scanned positions of the receiving antenna. It is clear from the figure that the tumor signal becomes amplified and so very identifiable.

## 6. Conclusions

A system of UWB breast tumor detection is designed. It consists of ultrashort monocycle pulse generator, transmitter and receiver antennas, and breast with and without tumor model. The transmitter employs an impulse-generator network to achieve less distortion, low ringing, and low power consumption. It produces monocycle pulses with 235 ps pulse duration and about 600 mV peak-to-peak voltage. The waveform produced by this transmitter is characterized by low ringing level and good balance between the positive and negative parts.

Antipodal Vivaldi antenna with an extremely wide −10 dB return loss bandwidth from 2.3 GHz to more than 11 GHz has been developed. This antenna element features directive over the required ultra-wideband.

Many simulation experiments have been carried out to identify and maximize the tumor signal. It is found that one can achieve a tumor signal in the order of mill volts. One can multiply this signal by accumulating the tumor signal resulting from different scanning positions of the receiver antenna.

This work proves that one can build an effective breast tumor detector using the UWB pulse technique together with some signal processing.

It is intended to complete building the system in the future.

## Figures and Tables

**Table 1:** Process corner simulation.

Model parameter	FF	TT	SS
Voltage (V)	1.3	1.2	1.1
Temperature (C°)	−20	27	70
Pulse Amp. (P-P) mv	530	600	440
Pulse width (ps)	200	235	250

**Table 2:** Performance comparison with published results.

Work	Tech *μ*m	*V*_*PP*_ mv	*T*_0_ ns	*P*_DC_ mv
Our	0.13	600	0.235	0.024
[12]	0.13	530	1.8	0.031
[14]	0.9	660	0.375	19.8
[15]	0.18	500	0.16	—
[16]	0.18	115.2	0.47	0.244
